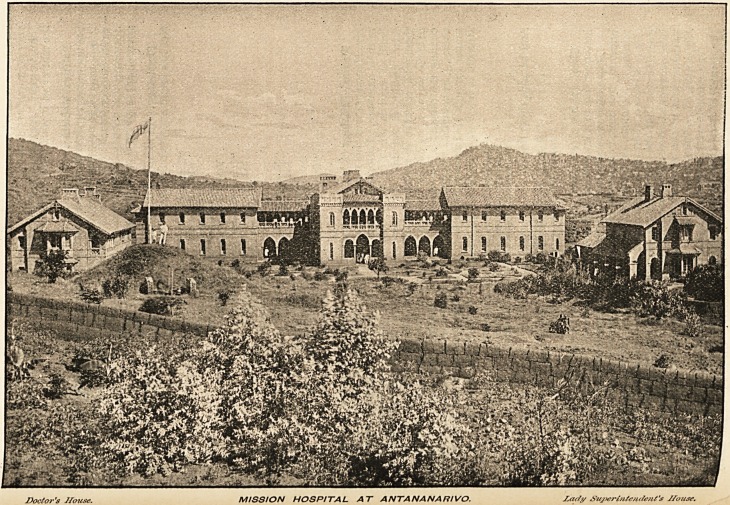# Scraps

**Published:** 1896-12

**Authors:** 


### SCRAPS

PICKED UP BY THE ASSISTANT-EDITOR.

**Medical "Bits of Old Bristol"** (I.).—In the *Journal* for June (p. 174) there was a reference to the accommodation for lepers in Bristol. It was stated on the authority of Dugdale that a Leper Hospital, dedicated to St. John Baptist, was built at Lawford's Gate on land given for the purpose by John, Earl of Moreton. John became specially connected with Bristol because his brother, Richard I., had, amongst other grants, conferred upon him the Earldoms of Gloucester and Somerset. The grant of land must have been between 1189, when John was made Earl of Moreton, and 1199 when he became King. In 1200 there was a direction issued (*Rotuli Chartarum*, ed. 1837, I. i. 77*b*) concerning the protection of the lepers of St. John's; but this is the only official reference to them I have been able to find. In 1208 John granted the charter (*Op. cit.*, 175*b*) which is printed in full in Dugdale (*Monasticon Anglicanum*, ed. 1830, VI. ii. 670), who gives "Lacford" where *Rot. Chart.* reads "Laffard." It says "hac cartâ nostrâ confirmâsse leprosis juxta Bristoll, croftam extra portam Lacford, in viâ versùs Bathoniam," but makes no mention of any dedication to St. John. A leper hospital which was dedicated to St. Laurence is mentioned in 1215 (*Rotuli Litterarum Clausarum*, ed. 1833, I. 227*a*) and in 8 Henry III. 1223-24 (*Calendarium Rotulorum Patentium*, ed. 1802, 13). In 32 Henry III., 1247-48, it is mentioned twice (*op. cit.*, 22) and in a charter granted in that year reference is made, "magistro et fratribus hospitalis leprosorum S. Laurentii in suburbio Bristolli," (Dugdale, *Op. cit.*, 671). There is evidently great uncertainty about the existence of two leper hospitals in that neighbourhood. Mr. John Latimer, who has kindly helped me in this investigation, tells me it is possible that in the 1200 direction a mistake occurred in the name of the hospital or that afterwards a change was made in the dedication. In Tanner's *Notitia Monastica* (ed. 1787) it is said that St. Laurence's leper hospital "was in Bell lane, near St. John's church," but this must be a confusion with the Church of St. Laurence which was in that situation and was pulled down in 1580. At no time could Bell Lane have been said to be "in suburbio Bristolli," and certainly no accommodation for lepers could have been in a spot so near the centre of the town. In connection with the so-called St. John's leper hospital, the records cited by Tanner have, with the exception of the 1200 direction, nothing to do with lepers, and in so far as they deal with any hospital of St. John they probably all refer to St. John's in Redcliff. Tanner also refers to William Wyrcestre (1415-1479), but these references (pp. 260, 274 of the 1778 edition of the *Itinerarium*) are to St. John's in Redcliff, which was not a leper hospital. Tanner has, in addition, a reference to Leland (about 1538), whose mention, however, is of "S. John's by

Radeclife" (*Itinerary*, ed. Hearne, 1769, VII., 92). This confusion is continued in *Bristol Past and Present*, 1881 (vol. II., Index and pp. 15, 115).

The Hospital of St. Laurence was given by Edward III. to the College of Westbury (Atkyns, *The Ancient and Present State of Glocestershire*, 2nd ed., 1768, p. 421). In 13 Henry IV., 1411-12, there is a mention of a hospital "de Laffordesyarde in suburbio Bristoll'" (*Cal. Rot. Pat.*, 258*b*), which probably refers to that of St. Laurence, although no specific title is given. At the dissolution about 1535 the hospital, as part of the College property, was granted to Sir Ralph Sadler, when, of course, it ceased to be an asylum for lepers. Sadler was in high favour at Court, and when Elizabeth came to Bristol in 1574 "she first alighted at St. Laurence Hospital, changed some apparel and was met at Lawford's Gate by the Mayor and Council" (Evans's *Chronological Outline*). In 1629 it had become with its grounds of 205 acres the Manor House belonging to Robert Hooke, one of a celebrated Bristol family, a member of which gave his name to "Hook's Mills," where the Orphan House at the bottom of Ashley Hill now stands. According to Barrett the site of St. Laurence's Hospital "abutted south on London highway and Chapel-lane on the East." No remains of it are now known, but the name is perpetuated in Lawrence Hill.

William Wyrcestre seems to be the sole authority on St. Mary Magdalene's Leper Hospital. He refers to it twice: "Capella Sanctæ Mariæ Magdalenæ ab antiquo fundata cum hospitali gentium leprosarum scita est in parte boriali versus pontem de Bryghtbow ultra domum capellanorum Willelmi Canyngys" (*Op. cit.*, p. 206); "Ecclesia hospitalis domus Sanctæ Mariæ Magdalenæ leprosorum in occidentali parte de Radclyffe-hylle in boriali parte viæ ad pontem Bryghtbow" (*Op. cit.*, p. 260).

No one who wishes to learn something about the mediæval leper hospital, which had more the character of a refuge or an asylum than of a place for medical treatment, should omit to give close attention to Simpson's articles on the subject in the *Edinburgh Medical and Surgical Journal* (Oct., 1841, Jan., April, 1842).

It would be pleasant if we could believe there was a particle of truth in "The Rolle of Seyncte Bartholemeweis Priorie," which Chatterton professed to have found among the Rowley papers and which the credulous Barrett (*History of Bristol*, p. 428) accepted as genuine. It is stated in it that "Atte the ende of the courte ys the Lazarre howse for thylke who havethe the leprous brennynge," and that provision was made "toe shryve the leperes wythe 10 markys bie the yeere to a fadre of the blacke frierie to shrive the lepeirys and 50 markys in lyke tyme to dresse ande docke theyre sorres, sayinge, lette us cure both spryte & bodye. From the yate we passe toe the ache chambre where attendeth foure mastre barboure surgeonnes under the behylte of the Austynian Frere." The library is said to have contained "Gylbertines rolle of Ypocrates:—The same fryarres booke of brennynge, Johan Stowe of the cure of mormalles & the waterie leprosie—the rolle of the blacke mainger: F. Lewis a Wodefordes booke of ailes." It is added: "of the reste of the Lazar house bee cellis & beddis for the Lazars, beeynge manie in number, the onlie roome else ys the halle where the pryoure summoneth councel of Bredrenne of physique blacke whyte grey & odhers: whanne some doughtie worke ys to bee donne on a Lazar, and the mastre barber surgeonne recevyth theyre order, the fryeres havethe for attendance iij groates fothe syttynge as was lefte bie the wordhie knyghte Syr Johan Somerville—leste hurte ande scathe bee done to the lepers, the whych mote bee avoyded; the sayings & notises of freeres bee wrote yn a rolle from the whych the barbour surgeonnes learn muche ande none botte those of Seynte Bartlemews maye loke thereynne: by whych meanes the barboure surgeonis wyll bee servytours there wythoute paye to gayne knowleche of aylimentes & theyr trew curis."

If the fanciful pseudo-antique spelling of this document and other suspicious things about it had not been enough to prove its falseness, the reference to "the rolle of the blacke mainger" should have been conclusive. The citation of the "blacke mainger," evidently thought by Chatterton to be some dire form of disease, is amusing. Barrett, in a serious note, illustrates it by quoting Chaucer, "on his skin a mormalle had he & a blacke manger." This misquoted reference is to Chaucer's Cook (Prologue, 386–7). Even without his surgical knowledge, Barrett ought not to have made this ludicrous mistake. After giving a description of the Cook's prowess in his particular business, Chaucer says:

> "But greet harm was it, as it thoughte me,
> That on his shine a mormal hadde he;
> For blankmanger, that made he with the beste."

Blankmanger, different in composition from the blancmange of to-day, was a well-known Middle-English dish and is frequently mentioned in contemporary books, in which its ingredients and mode of preparation are given. It appears in the *Catholicon* as "Blawemanger," and several other references to it will be found in Mr. Herrtage's note to it in the Camden Society edition. Were it not that no literary crime can be pardoned we might, for the exquisite drollery of its blunder, be tempted to forgive the Chatterton-Barrett conversion of the appetising "blankmanger" into a disease, "blacke mainger," presumably a sort of "Black Death."

Mormal was well known as a term for an intractable sore and derived its name from *malum mortuum*.

In 1528 the buildings of St. Bartholomew's Priory, which gave Chatterton this splendid opportunity, were transferred to the Mayor and Burgesses for the purposes of a Grammar School founded under a bequest from Robert Thorne. It continued to be used as such till 1769, when an exchange was effected with Queen Elizabeth's Hospital, now better known as the City School, which was then carried on in premises occupying the site of the present Merchant Venturers' College. Queen Elizabeth's Hospital, which had been founded under the will of John Carr in 1586 as a school for boys upon the plan of Christ's Hospital, remained in St. Bartholomew's till 1847, when it was removed to its present fine building on Brandon Hill, In 1879 the Grammar School was removed to the buildings in Tyndall's Park. Some remains of St. Bartholomew's Priory still exist near the bottom of Christmas Steps, and on the western side of the doorway can be seen the mutilated figure of the Virgin and Child.

**Medical Missions.**—I take the following notes from the November Quarterly Paper of the Edinburgh Medical Missionary Society. When the victorious French troops entered Antananarivo it was thought that the disturbed state of Madagascar would soon be settled. But the whole country, with the exception of the capital and a few garrisoned towns, was in a state of ferment and lawlessness. Two of Madagascar's most devoted missionaries, with their little girl, have been cruelly murdered, and others have had to fly from their stations to save precious life. The flourishing Norwegian Mission Station at Antsirabe, with its medical mission hospital and leper village, have been utterly destroyed. Hundreds of churches have been burnt or razed to the ground, and teachers, evangelists, and pastors hounded from place to place. The medical missionary hospital at Antananarivo[1] is one of the best equipped mission hospitals in the world, though not by any means the largest. It was built chiefly by the Friends' Foreign Mission Association, and is maintained by that mission and the London Missionary Society. On Sept. 30th, 1895, the Capital was attacked, and for some time the hospital was in considerable danger. One shell burst just in front of the hospital, but no one was injured. When the surrender of the town took place the hospital staff were very busy in attending to the wounded, and there the French and Malagasy were first gathered and reconciled under one roof. The French doctors praised the well-trained native nurses highly. Soon after General Duchesne's return to Paris, the French Government granted Miss Byam, the lady superintendent, a medal of honour in acknowledgment of her devotion and help.

[1] The view on the opposite page I owe to the kindness of Dr. Sargood Fry, who will, I am sure, be quite ready to give information about, or receive contributions to, the Edinburgh Medical Missionary Society, of which he is the secretary and superintendent, and whose office is at 56 George Square, Edinburgh.

*Doctor's House.* MISSION HOSPITAL AT ANTANANARIVO. *Lady Superintendent's House.*

**Clinical Records** (17).—At an East-end Hospital a man was brought in terribly smashed. A new house surgeon having examined him said to his wife, "I fear your poor husband is dead." "No, I ain't," said the supposed corpse. "Hush, John, be quiet," said the wife, "the gentleman must know better than you what 's the matter with you."—*St. Thomas's Hospital Gazette.*

**Political Medicine.**—As there seems to be some indication that the Venezuelans are not quite prepared to consider that their difficulty with England is ended, it is of interest to note that the *Medical Age* says that "An Up North Democrat wrote Senator Roach: 'All the fools around here are talking about the Munroe doctoring, and nobody knows what it is, and I don't know it myself; but if the government is giving it away send me what you can.'"

**Physiology.**—In some American papers recently a well-known physiological fact has found expression in the following Rondeau:—

"I can't conceive," she archly cried,
"Wherein you men can longer pride
Yourselves from female rivals free,
For surely we have grown to be
Your peers in ev'ry human stride.

"It is a truth that none dare hide;
Yet why you men will not decide
To recognise the new decree
I can't conceive.

"Now, *entre nous*, won't you confide
And tell me true, all jokes aside,
What difference the world can see
Between your manly self and me?"
"To tell you truly," he replied,
"I can't conceive."

**Medical Philology** (XX.).—The *Catholicon* has "Coleryke; *colera; coleric*us." Mr. Herrtage gives this note: "Men were divided into four classes, according to their humours. Laurens Andrewe says, in his *Noble Lyfe* [1510], 'And the bodij of man is made of many diuers sortes of ly*m*mes as senewes, vaynes, fatte, flesshe, & skynne. And also of the foure moistours, as sanguyne, flematyke, coleryke & melancoly' (fol. a iv. back. col. 2). Men die, he says, in three ways: 1, by one of the four elements of which they are made, overcoming the others; 2, by *humidum radicale*, or 'naturall moystour,' forsaking them; 3, by wounds—'the coleryke commeth oftentymes to dethe be accedentall maner through his hastines, for he is of nature hot and drye.' So also John Russell, in his *Boke of Nurture* [*c.* 1460] (Babees Boke, p. 53), says:—

'The second course *colericus* by callynge
Fulle of Fyghtynge blasfemynge, & brallynge,
Fallynge at vergaunce with felow and frie.'

And he adds these lines—

Colericus:

*Hirsutus, Fallax, irascens, prodigus, satis audax,*

*Astutus, gracilis, siccus, croceique coloris.*"

Skeat in his *Etymological Dictionary* says: "The *h* is a 16th century insertion, due to a knowledge of the source of the word."

Hippocrates is usually credited with being the author of the theory of the humours, but there is much doubt about the authenticity of the treatise containing it, although, as Rutherford Russell points out (*History and Heroes of the Art of Medicine*), there is evidence that his practice was affected by it. The doctrine was that the four humours, namely, blood, phlegm, yellow and black bile, occasioned diseases by the prevalence of one or other of them, according to the seasons of the year and other circumstances (see *The Genuine Works of Hippocrates*, ed. Adams, 1849, *passim*).

The choleric were subject to special spiritual temptation if the writer of the *Ayenbite of Inwyt* (1240), referred to by Mr. Herrtage and quoted in the *New English Dictionary*, is to be accepted as an authority. On p. 157 of the Early English Text Society edition of that work it is said that "þe dyevel . . . asayleþ stranglakest . . . þane colrik mid ire and mid discord."

In line 3 of the quotation from the *Boke of Nurture* for "*vergaunce*" read "*veryaunce*," and for "*frie*" read "*fere*."

## Figures and Tables

**Figure f1:**